# Conserved and non-conserved characteristics of porcine glial cell line-derived neurotrophic factor expressed in the testis

**DOI:** 10.1038/s41598-018-25924-5

**Published:** 2018-05-16

**Authors:** Kazue Kakiuchi, Kazumi Taniguchi, Hiroshi Kubota

**Affiliations:** 1 Laboratory of Cell and Molecular Biology, School of Veterinary Medicine, Kitasato University, Towada, Aomori, Japan; 2 Laboratory of Veterinary Anatomy, School of Veterinary Medicine, Kitasato University, Towada, Aomori, Japan

## Abstract

Glial cell line-derived neurotrophic factor (GDNF) is essential for the self-renewal and proliferation of spermatogonial stem cells (SSCs) in mice, rats, and rabbits. Although the key extrinsic factors essential for spermatogonial proliferation in other mammals have not been determined, GDNF is one of the potential candidates. In this study, we isolated porcine GDNF (pGDNF) cDNAs from neonatal testis and generated recombinant pGDNF to investigate its biological activity on gonocytes/undifferentiated spermatogonia, including SSCs. In porcine testis, long and short forms of GDNF transcripts, the counterparts of pre-(α)pro and pre-(β)pro GDNF identified in humans and rodents, were expressed. The two transcripts encode identical mature proteins. Recombinant pGDNF supported proliferation of murine SSCs in culture, and their stem cell activity was confirmed by a transplantation assay. Subsequently, porcine gonocytes/undifferentiated spermatogonia were cultured with pGDNF; however, pGDNF did not affect their proliferation. Furthermore, GDNF expression was localised to the vascular smooth muscle cells, and its cognate receptor GFRA1 expression was negligible during spermatogonial proliferation in the testes. These results indicate that although pGDNF retains structural similarity with those of other mammals and conserves the biological activity on the self-renewal of murine SSCs, porcine SSCs likely require extrinsic factors other than GDNF for their proliferation.

## Introduction

Glial cell line-derived neurotrophic factor (GDNF), a distant member of the TGFß superfamily, was originally discovered as a survival factor for dopaminergic neurons^1^. Although GDNF is found to be expressed throughout the central nervous system during development as well as in the adult brain, subsequent studies have revealed that this factor is widely expressed in various non-neuronal tissues, including the embryonic kidney, gastrointestinal tract, and testis^2^. In GDNF-knockout mice, embryonic development of the enteric nervous system and kidney has been shown to be severely impaired, resulting in neonatal deaths^3–5^. Although homozygous GDNF-knockout mice die within 24 h after birth, heterozygous GDNF-deficient mice survive with some abnormalities. In the testes of heterozygous mutant mice, spermatogonial proliferation is reduced and spermatogenesis is eventually abolished in most seminiferous tubules, which results in a Sertoli cell only-phenotype^6^. Conversely, GDNF-overexpressing transgenic mice showed an abnormal accumulation of spermatogonia. The phenotype was attributed to the blockade of differentiation of undifferentiated spermatogonia^6^.

In mice, spermatogenesis starts shortly after birth and continues throughout adult life. Continuous sperm production depends on the capacity of spermatogonial stem cells (SSCs) to self-renew and constantly generate committed spermatogonia that eventually differentiate into sperm. Although the number of SSCs in the adult testis is extremely low, which is estimated to be around 0.03% of the total cell population^7^, murine SSCs can be identified unequivocally by transplantation into the seminiferous tubules of spermatogenesis-abrogated infertile mice^8,9^. Following transplantation, only SSCs can colonize on the basement membrane of the recipient seminiferous tubules and reconstitute continuous spermatogenesis. Although previous mice studies involving GDNF knockout and overexpression have strongly suggested that GDNF regulates SSC self-renewal, a definitive proof showing that GDNF is an essential exogenous factor required for the self-renewing proliferation of SSCs was demonstrated by an *in vitro* study using a defined condition in conjunction with the transplantation assay^10,11^. Using a serum-free defined medium, the study clearly demonstrated that the continuous proliferation of murine SSCs, resulting in the reconstitution of spermatogenesis in the recipient mouse testes after transplantation, is strictly dependent on GDNF^11^.

Reconstitution of xenogeneic spermatogenesis in recipient mouse testis has been shown to successfully occur when SSCs from rats or hamsters are transplanted^12,13^. These findings demonstrate that the factors involved in spermatogonial proliferation and differentiation are conserved among rodents. In fact, rat SSCs continuously proliferate in the presence of GDNF in the serum-free culture system developed for mouse SSCs^14^. On the other hand, when testis cells from non-rodents such as domestic animals and primates were introduced into recipient mouse testes, colonization and proliferation of spermatogonia were observed, however no donor-derived spermatogenesis was reconstituted^15–18^. These results suggested that exogenous factors for spermatogonial proliferation are conserved between mouse and non-rodent mammalian species, but differentiation factors are species-specific. The efficiency of colonization and proliferation of xenogeneic spermatogonia in the mouse seminiferous tubules varied in each species examined. In some species, such as rabbit and pig, the proliferation of spermatogonia in mouse testes continued for several months^15,16^. As predicted, serum-free culture experiments have demonstrated that GDNF plays a critical role in the unlimited proliferation of rabbit undifferentiated spermatogonia^19^. Following transplantation into immunocompromised mouse testes, the cultured rabbit undifferentiated spermatogonia were shown to colonize in the recipient seminiferous tubules and developed clusters of spermatogonia, which retained the original phenotype and maintained at least for six months^19^. These results indicate that self-renewing proliferation of rabbit undifferentiated spermatogonia requires GDNF and suggest that they likely contained SSCs because of their long-lived nature. However, a functional assay for rabbit SSCs, in which both self-renewal and differentiation can be evaluated, has not been developed; thus, their stem cell activity remains to be confirmed.

Studying non-rodent-derived SSCs is important for understanding the regulatory mechanism of self-renewal, the process of spermatogenesis, and the maintenance of male germline for application in comparative biology and biomedical sciences. Furthermore, SSCs can be used for the generation of genetically modified offspring^20,21^. Pig is a valuable domestic animal, and porcine SSCs hold great promise in the fields of agriculture and medicine. However, because porcine SSCs have not yet been identified, their biology remains largely unknown. Our preliminary culture experiments indicated that human GDNF (hGDNF), which has been used for rodent and rabbit SSC cultures^11,14,19^, did not stimulate proliferation of porcine gonocytes (also called prospermatogonia or prespermatogonia^22,23^)/undifferentiated spermatogonia, suggesting that the biological function of GDNF on spermatogonia may not be conserved between pigs and rodents/humans. Therefore, in this study, we evaluated the proliferative activity of porcine GDNF (pGDNF) on SSCs and its role in spermatogonial proliferation in testes. To achieve this, we cloned the pGDNF cDNA and generated recombinant proteins to investigate their biochemical characteristics and biological activity on murine SSCs and porcine gonocytes/undifferentiated spermatogonia. Furthermore, expression of pGDNF in postnatal testes was investigated to evaluate its role in spermatogonial proliferation in the testes.

## Results

### Molecular cloning and identification of pGDNF cDNAs

**Figure 1:**
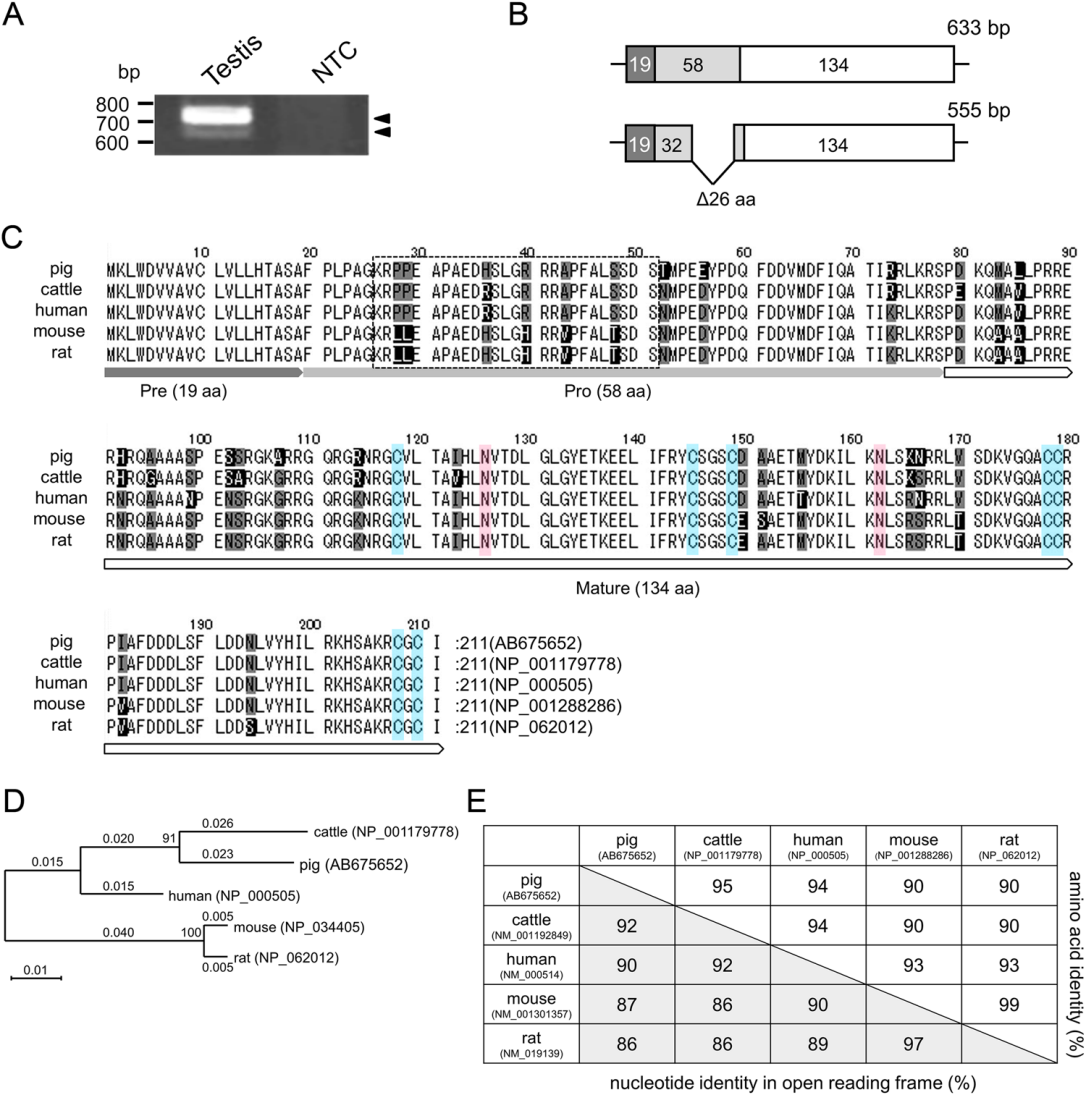
Molecular cloning of pGDNF cDNA. (**A**) RT-PCR analysis of pGDNF. Two GDNF transcripts (0.7 kb and 0.65 kb) were identified in the newborn testis. NTC: no template control. (**B**) The protein coding regions of the long and short transcripts of pGDNF. Dark grey, light grey and white boxes indicate the pre-regions, pro-regions and mature regions, respectively. The numbers of amino acids in each region are shown. The short transcript lacks 26 amino acids. (**C**) Alignment of pGDNF amino acid sequence with those from other mammalian species. The amino acids that are not identical in 3 to 5 sequences are shaded black, and those that are identical in 3 to 4 sequences are shaded grey. The 26 amino acids lacking in the short transcript are boxed. Because the short forms are produced by alternative splicing, glycine^25^ is substituted with alanine^25^ at the joining site. The short transcript of bovine GDNF has not been identified. The seven conserved cysteine residues in the TGFß superfamily are indicated in blue. The *N*-linked glycosylation sites are indicated in red. (**D**) Phylogenetic presentation of pGDNF proteins. Amino acid sequences were aligned using Clustal W. (**E**) The percent identity of GDNF nucleotide and amino acid sequences between five mammalian species.

Because only partial pGDNF mRNA sequences have been reported in the database (DDBJ/GenBank/EMBL), we initially determined the complete coding sequence of pGDNF cDNA (refer to the Materials and Methods section). Reverse transcriptase PCR (RT-PCR) of the porcine neonatal testis cDNA generated two amplified fragments of 0.7 kb and 0.65 kb (Fig. 1A). The longer fragment was dominantly expressed in the testis. Both the PCR products were cloned into plasmid vectors and the nucleotide sequences were determined. The longer transcript contained a 633-bp open reading frame (ORF) and the shorter transcript contained a 555-bp ORF, which encode 211 and 185 amino acids, respectively (DDBJ/GenBank/EMBL accession nos. AB675652 and AB675653) (Fig. 1B). GDNF is synthesised in the form of precursor pre-pro GDNF which is proteolytically cleaved to become the mature form^1^. In human, rat, and mouse, long and short forms of GDNF mRNA are expressed, and they encode pre-(α)pro GDNF and pre-(β)pro GDNF, respectively^24–28^. Although pre-(α)pro GDNF is 26 amino acids longer than pre-(β)pro GDNF, the two transcripts produce an identical mature GDNF after post-translational processing. This is because the pre-(β)pro pGDNF is generated by alternative splicing of the exon encoding the pro-domain and thereby shorten 26 amino acids of pro-domain of the pre-(α)pro pGDNF. Compared with the human and rodent GDNF cDNA sequences, the two forms of pGDNF cDNAs identified in the pig testis correspond to pre-(α)pro GDNF and pre-(β)pro GDNF (Fig. 1B). Comparison of the amino acid sequences of pre-(α)pro GDNF between pig and other mammals is indicated in Fig. 1C. Two potential *N*-linked glycosylation sites and the positions of seven cysteine residues, which are conserved in all mature GDNFs identified, were found in the pGDNF cDNA sequences (Fig. 1C). The amino acid sequence of pGDNF showed high homology to that of bovine GDNF (95%) followed by hGDNF (94%), and mouse and rat GDNFs (90%) (Fig. 1D,E).

### Characterization of recombinant pGDNF

**Figure 2:**
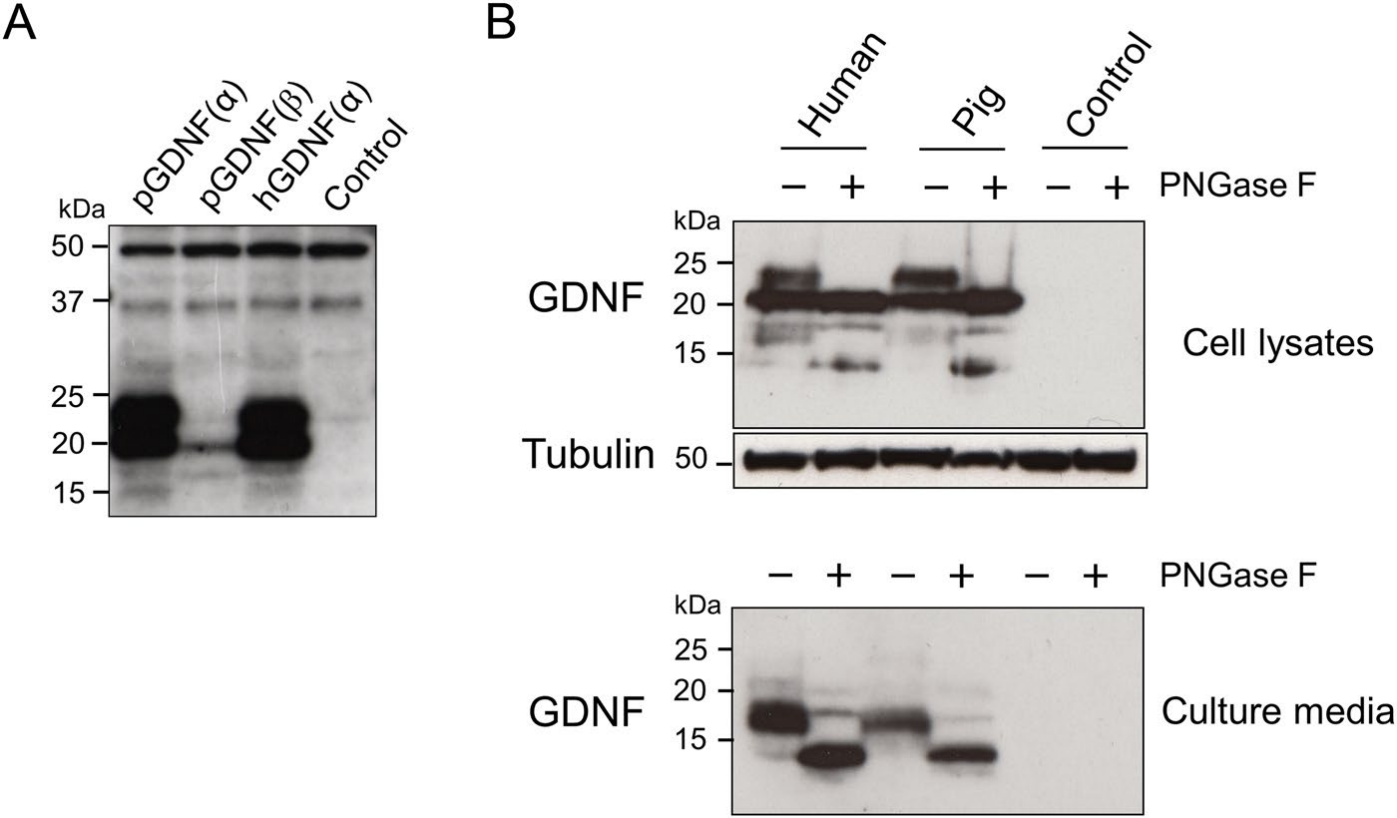
Expression analysis of recombinant pGDNF. (**A**) Western blot analysis of COS-1 cells transfected with pre-(α)pro pGDNF, pre-(β)pro pGDNF, or pre-(α)pro hGDNF expression plasmid. The control was transfected with sterile water as a mock transfection. Two GDNF-specific bands (24 kDa and 20 kDa) were detected in the cell lysates of pre-(α)pro GDNF-transfected COS-1 cells, while a single weak band (20 kDa) was detected in the cell lysates of pre-(β)pro pGDNF-transfected COS-1 cells. Non-specific bands of 50 kDa and 35 kDa were detected in all samples. (**B**) Western blot analysis of cell lysates and culture medium from COS-1 cells transfected with pre-(α)pro GDNF or mock transfected cells. The samples were treated (+) or untreated (−) with PNGase F that removes *N*-linked glycans. Tubulin expression was used as an internal control for cell lysate samples. The culture media (10 μL) of transfected COS-1 cells were used in each sample.

In order to investigate pGDNF at the protein level, pre-(α)pro pGDNF and pre-(β)pro pGDNF cDNAs were transfected into COS-1 cells to express recombinant proteins. Pre-(α)pro hGDNF cDNA was also transfected for comparison. Strong 24 kDa and 20 kDa bands of GDNF from the lysates of pre-(α)pro pGDNF and pre-(α)pro hGDNF transfected cells were detected by western blot analysis, while the cell lysate of pre-(β)pro pGDNF showed only a weak 20 kDa band (Fig. 2A). A previous study showed a similar expression pattern, in which GDNF expression in cells transfected with pre-(β)pro hGDNF cDNA was weaker than in those transfected with pre-(α)pro hGDNF cDNA^29,30^. Hence, we only used the pre-(α)pro GDNF cDNA for further experiments. The 24 kDa and 20 kDa bands detected (Fig. 2A) are presumed to correspond to glycosylated and unglycosylated pro-GDNF^1,29,30^. To confirm *N*-glycosylation and investigate the expression of the mature GDNF in the culture medium, we transfected pre-(α)pro pGDNF and pre-(α)pro hGDNF cDNAs into COS-1 cells, and the supernatants and the transfected cells were separately collected. Subsequently, both samples were treated with peptide-*N*-glycosidase F (PNGase F). As predicted, western blot analysis showed that the 24 kDa band disappeared after treatment with PNGase F in the cell lysates (Fig. 2B). The glycosylated and unglycosylated mature forms of pGDNF in culture medium were 17 kDa and 13 kDa, respectively (Fig. 2B). Overall, the molecular forms in the culture medium and cell lysates of transfected COS-1 cells were indistinguishable between pGDNF and hGDNF. Collectively, the biochemical characteristics of the recombinant products of pre-(α)pro pGDNF and pre-(α)pro hGDNF were essentially identical, indicating that the post-translational modification of GDNF was conserved between pig and human.

### Evaluation of the biological activity of pGDNF on murine SSCs

**Figure 3:**
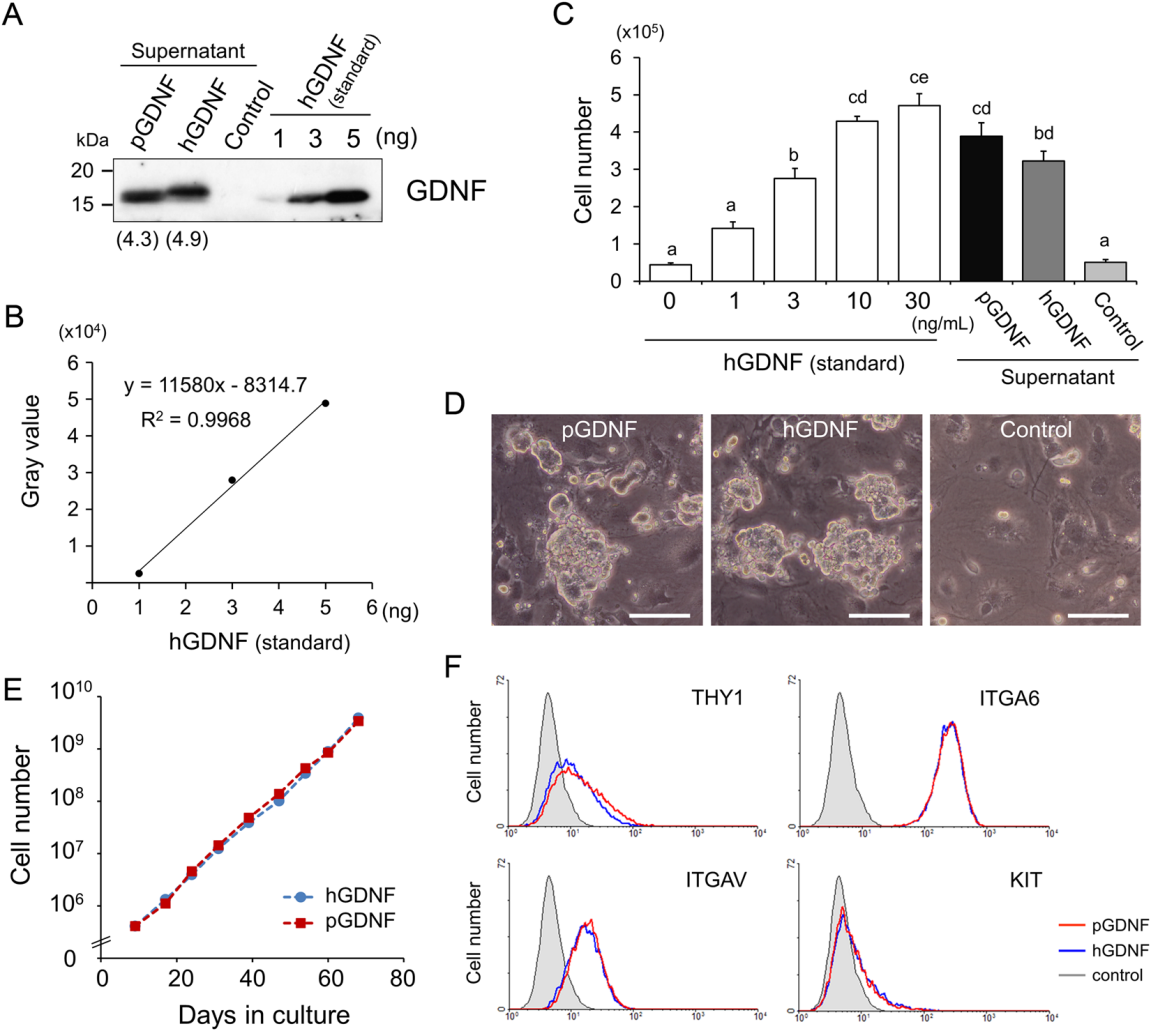
Evaluation of the proliferative activity of recombinant pGDNF on murine SSCs. (**A**,**B**) Measurement of GDNF concentration in the culture media of pGDNF-293 and hGDNF-293 cells by western blot analysis. The culture media (10 μL) of pGDNF-293, hGDNF-293 and non-transfected 293 cells and purified recombinant hGDNF (1, 3 and 5 ng) for preparing a calibration curve were electrophoresed. A calibration curve was prepared by the gray values of the purified hGDNF. The amounts of GDNF in the culture media of pGDNF-293 and hGDNF-293 were calculated to be 4.3 and 4.9 ng, respectively. (**C**) Proliferation of mouse clump-forming germ cells enriched for SSCs by pGDNF. Cells were seeded at 2 × 10^5^ cells/mL/well in a 24-well plate and cultured with pGDNF (supernatant), hGDNF (supernatant) or purified recombinant hGDNF (standard, 1, 3, 10 and 30 ng/mL) for 7 days. The supernatants (30 μL) of pGDNF-293, hGDNF-293 or non-transfected 293 cells were added to 1 mL of SFM supplemented with FGF2. Data are shown as mean ± SEM (n = 3). Different letters indicate significant differences (P < 0.01 except for a vs. b = P < 0.05). (**D**) Phase contrast images of mouse SSCs cultured with pGDNF or purified recombinant hGDNF. Mouse SSCs formed cellular clumps and proliferated in the presence of GDNF, while no cell proliferation was observed in the absence of GDNF. Bars = 100 µm. (**E**) Initiation and continuous proliferation of germ cell clumps by pGDNF. The cell numbers of clump-forming germ cells cultured from freshly isolated testis cells with pGDNF or purified recombinant hGDNF are indicated. (**F**) Antigenic profile of clump-forming germ cells cultured with pGDNF or hGDNF. The surface phenotypes of cells cultured in either condition were THY1^lo^ ITGA6^+^ ITGAV^lo^ KIT^−^.

We further evaluated the biological activity of pGDNF on murine SSCs. For this purpose, we established two stable cell lines, pGDNF-293 and hGDNF-293, from HEK293 cells transfected with pEPI-CAG-pGDNF or pEPI-CAG-hGDNF, respectively. To evaluate the amount of GDNF in the supernatants, culture medium from each cell line was collected and subjected to western blot analysis. A 17 kDa signal corresponding to the glycosylated form of GDNF was detected in the culture media of both pGDNF and hGDNF cell lines (Fig. 3A). The concentration of pGDNF and hGDNF in each culture supernatant was evaluated as 4.3 or 4.9 ng per 10 μL, respectively (Fig. 3B).

In the presence of hGDNF, under serum-free culture conditions, murine undifferentiated spermatogonia, including SSCs form cellular clumps and proliferate continuously^11,31^. To assess the biological activity of pGDNF on the proliferation of murine SSCs, the clump-forming germ cells from neonatal C57BL/6-Tg (CAG-EGFP) mice (hereafter referred to as GFP mice) were cultured with the pGDNF-293 supernatant. In the presence of 30 μL/mL of supernatant (12.9 ng/mL of GDNF), the number of clump-forming cells increased by 2.5-fold in one week, whereas the control supernatant from HEK293 cells did not support cell proliferation (Fig. 3C,D). Compared with the standard hGDNF samples (purified recombinant hGDNF, R&D systems), the proliferative activity of pGDNF-293 supernatant was equivalent to 10 ng/mL (Fig. 3C,D). The proliferative activity of hGDNF-293 supernatant (14.7 ng/mL of hGDNF) was similar to that of pGDNF-293 supernatant (Fig. 3C). Furthermore, pGDNF-293 supernatant could initiate germ cell clump-formation from freshly isolated testis cells and support continuous proliferation for two months at the same rate as standard hGDNF (Fig. 3E). The antigenic profile of the clump-forming germ cells cultured with pGDNF or hGDNF for two months was THY1^lo^ ITGA6^+^ ITGAV^lo^ KIT^−^ (Fig. 3F), which was identical to that of SSCs in mouse testes^10,32^.

**Figure 4:**
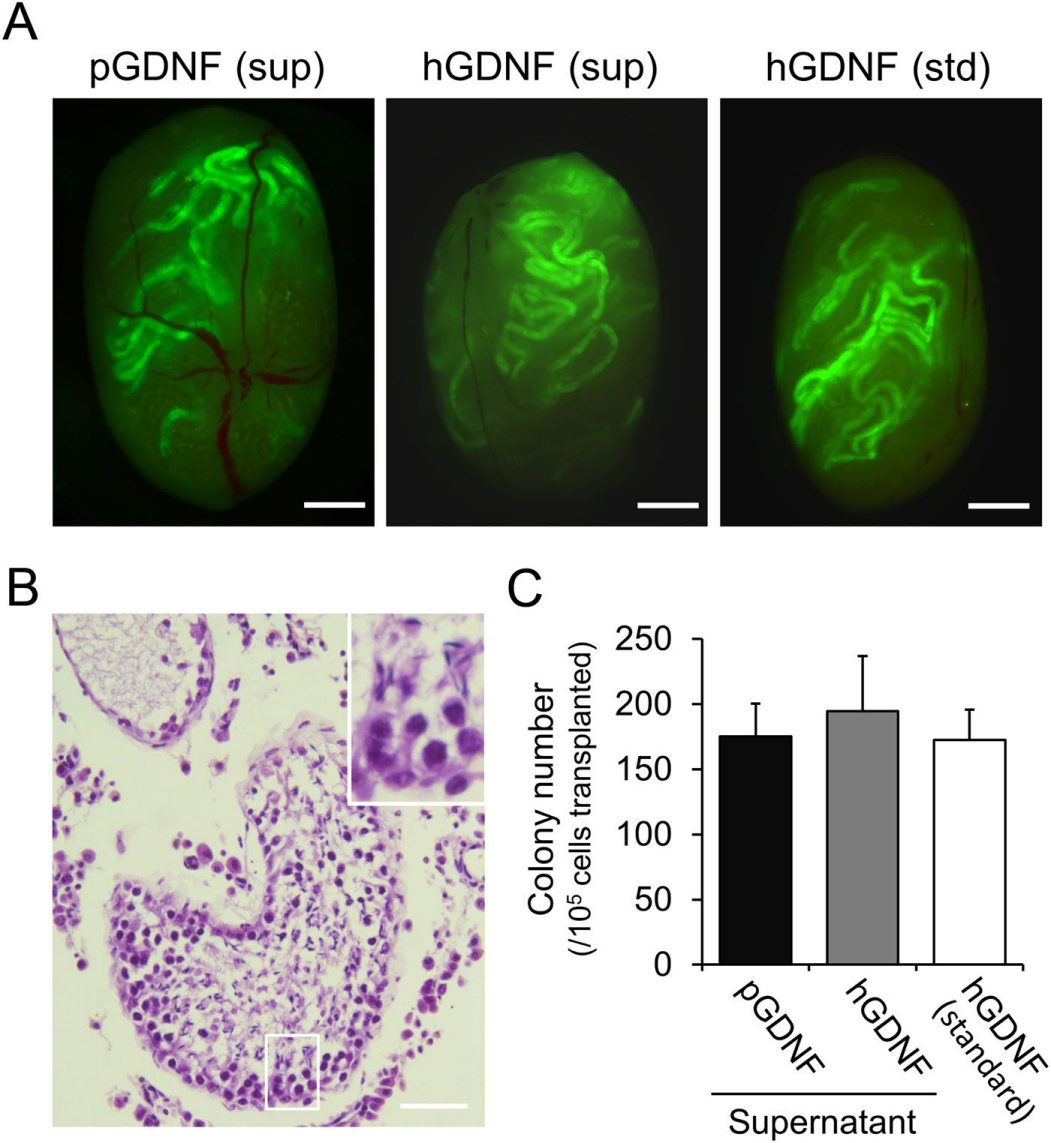
Functional transplantation assay of SSCs cultured with pGDNF. (**A**) Fluorescent images of transplanted testes. Clump-forming germ cells derived from GFP mice were expanded in the presence of pGDNF or hGDNF for 2 weeks following transplantation. Purified recombinant hGDNF was used as a control. Bars = 1 mm. sup, supernatant; std, standard. (**B**) Histological analysis of transplanted testes. A hematoxylin and eosin stained cross-section of recipient testes transplanted with clump-forming germ cells cultured with pGDNF is shown. Elongated spermatids were observed (inset). Bar = 50 µm. (**C**) Spermatogenic colony number per 10^5^ cells transplanted. Culture supernatants from pGDNF-293 and hGDNF-293, and standard hGDNF showed an equivalent effect on the proliferative activity of murine SSCs. Data are shown as mean ± SEM, and 20, 29 and 17 recipient testes were analysed for pGDNF-293, hGDNF-293 and standard hGDNF, respectively.

To determine the stem cell activity of the germ cell clumps cultured with pGDNF, a functional transplantation assay for SSCs was carried out. Following transplantation into infertile mouse testes, donor SSCs reconstituted spermatogenic colonies in the recipient testes^8,9^, and in previous studies, approximately 200 spermatogenic colonies per 10^5^ THY1^lo^ ITGA6^+^ spermatogonia transplanted were generated^10,11^. Two weeks after culture with pGDNF or hGDNF, germ cell clumps derived from GFP mice were transplanted into the seminiferous tubules of Busulfan-treated infertile recipient mice. Recipient testes were analysed at two months after transplantation (Fig. 4A,B), and the numbers of fluorescent spermatogenic colonies were counted^31^. Germ cell clumps cultured with the supernatants from pGDNF-293 and hGDNF-293, and standard hGDNF generated 175 ± 25 colonies (n = 20), 195 ± 42 colonies (n = 29) and 172 ± 23 colonies (n = 17) per 10^5^ cells transplanted, respectively (Fig. 4C). There were no significant differences in the SSC activity between these groups, demonstrating that pGDNF supports the self-renewal and proliferation of murine SSCs in the same way as hGDNF.

### Evaluation of the biological activity of pGDNF on porcine gonocytes/undifferentiated spermatogonia

**Figure 5:**
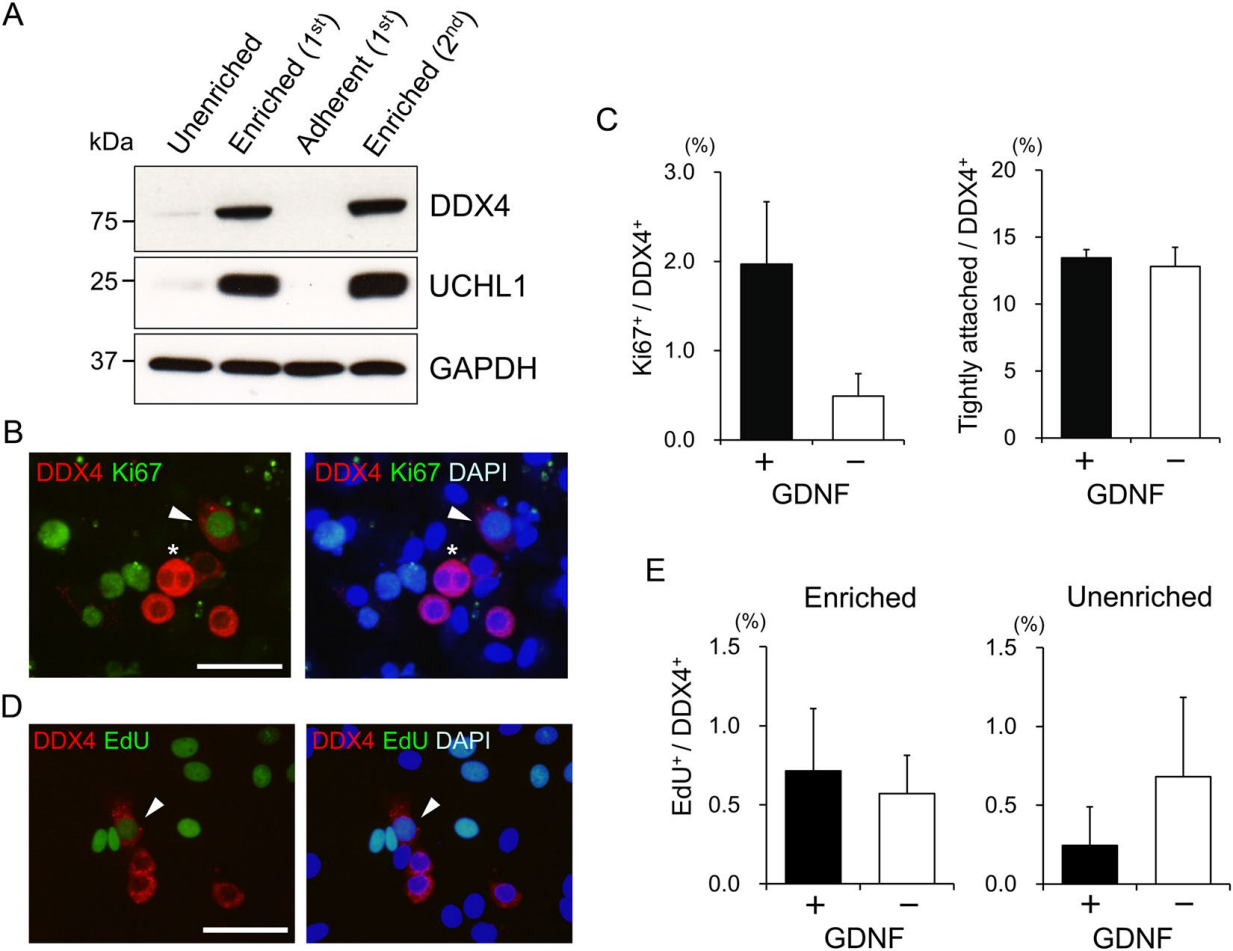
Evaluation of biological activity of pGDNF on the proliferation of porcine gonocytes/undifferentiated spermatogonia. (**A**) Enrichment of gonocytes/undifferentiated spermatogonia by differential plating. The first non-adherent (Enriched, 1^st^), the second non-adherent (Enriched, 2^nd^), and the first adherent cell fractions in addition to the unselected cell fraction (Unenriched) from porcine testicular cells were analysed by western blot analysis. DDX4 and UCHL1 were strongly expressed in the enriched fractions. (**B**) Immunofluorescence analysis of porcine gonocytes/undifferentiated spermatogonia cultured with pGDNF. A DDX4^+^ Ki67^+^ cell (arrow head) and tightly attached DDX4^+^ cells (asterisk) are indicated. Nuclei were counterstained with DAPI. Bar = 50 µm. (**C**) Effect of pGDNF on porcine gonocytes/undifferentiated spermatogonia. No significant difference was found between the number of DDX4^+^ Ki67^+^ or tightly attached DDX4^+^ cells in the presence and absence of pGDNF. Three independent experiments were performed. More than 100 DDX4^+^ cells were analysed in each experiment (mean ± SEM, n = 3). (**D**) EdU incorporation analysis for DNA replication in porcine gonocytes/undifferentiated spermatogonia cultured with pGDNF. A DDX4^+^ EdU^+^ cell (arrow head) is indicated. Nuclei were counterstained with DAPI. Bar = 50 µm. (**E**) Effect of pGDNF on enriched or unenriched porcine gonocytes/undifferentiated spermatogonia. No significant difference was found between the number of DDX4^+^ EdU^+^ cells in the presence and absence of pGDNF in either cell fraction. Four independent experiments were performed. More than 100 DDX4^+^ cells were analysed in each experiment (mean ± SEM, n = 4).

Next, we examined whether pGDNF stimulates proliferation of porcine gonocytes/undifferentiated spermatogonia. Porcine spermatogenesis starts approximately 16 weeks after birth^33^. From birth to the onset of spermatogenesis, gonocytes migrate to the basement membrane, turn into undifferentiated spermatogonia, and eventually give rise to SSCs^34–36^. Some gonocytes/undifferentiated spermatogonia have SSC activity as early as 10 days after birth^37^. To investigate the mitogenic effect of pGDNF, we cultured gonocytes/undifferentiated spermatogonia from 5–6-week-old porcine testes in the presence or absence of pGDNF. Before the onset of spermatogenesis, gonocytes/undifferentiated spermatogonia are the only germ cells in the postnatal testis, and they can be identified by the conserved germ cell marker DDX4 expression^34–36^. Although the percentage of gonocytes/undifferentiated spermatogonia was 1.8 ± 0.2% (n = 3) in fresh testicular cell suspensions, that was increased up to 9.0 ± 2.5% (n = 3) after two rounds of differential plating for their enrichment. Western blot analysis showed that the expression of DDX4 and another gonocyte/spermatogonia marker, UCHL1^35,36^, was accordingly increased in the enriched populations (Fig. 5A). After six days of culture with pGDNF, the enriched cells were analysed by immunocytochemistry for the expression of the proliferation marker Ki67 and DDX4 in order to identify proliferating gonocytes/undifferentiated spermatogonia (Fig. 5B). The immunocytochemistry indicated that Ki67^+^ DDX4^+^ cells were as few as 2.0 ± 0.7% and 0.5 ± 0.2% (n = 3) in the presence and absence of pGDNF, respectively (Fig. 5C). The number of Ki67^+^ DDX4^+^ cells was apparently low, and there was no significant difference between the two conditions (P = 0.12). In addition, the number of tightly attached germ cells, which represents an initial sign of clump formation in the culture, was equal in the two culture conditions (Fig. 5B,C). To confirm the results of the Ki67-immunocytochemical analysis, 5-ethynyl-2′-deoxyuridine (EdU) incorporation assay was performed. Enriched gonocytes/undifferentiated spermatogonia from 2–5-week-old porcine testes were cultured in the presence or absence of pGDNF for 6 days. The cultured cells were incorporated with 10 μM EdU for 24 h and stained with anti-DDX4 antibody to identify gonocytes/undifferentiated spermatogonia (Fig. 5D). The EdU incorporation assay indicated that the number of EdU^+^ DDX4^+^ cells was even lower and 0.7 ± 0.4% and 0.6 ± 0.2% (n = 4) in the presence and absence of pGDNF, respectively (Fig. 5E). Furthermore, the EdU incorporation assay was also performed for the unenriched testicular cells to avoid the effect of the initial selection process. Again, the percentage of EdU^+^ DDX4^+^ cells in the unenriched populations was as few as 0.2 ± 0.2% and 0.7 ± 0.5% (n = 4) in the presence and absence of pGDNF, respectively (Fig. 5E). These results indicated that pGDNF has little, if any, mitogenic effect on the porcine gonocytes/undifferentiated spermatogonia.

### Expression analysis of pGDNF in the porcine testes

**Figure 6:**
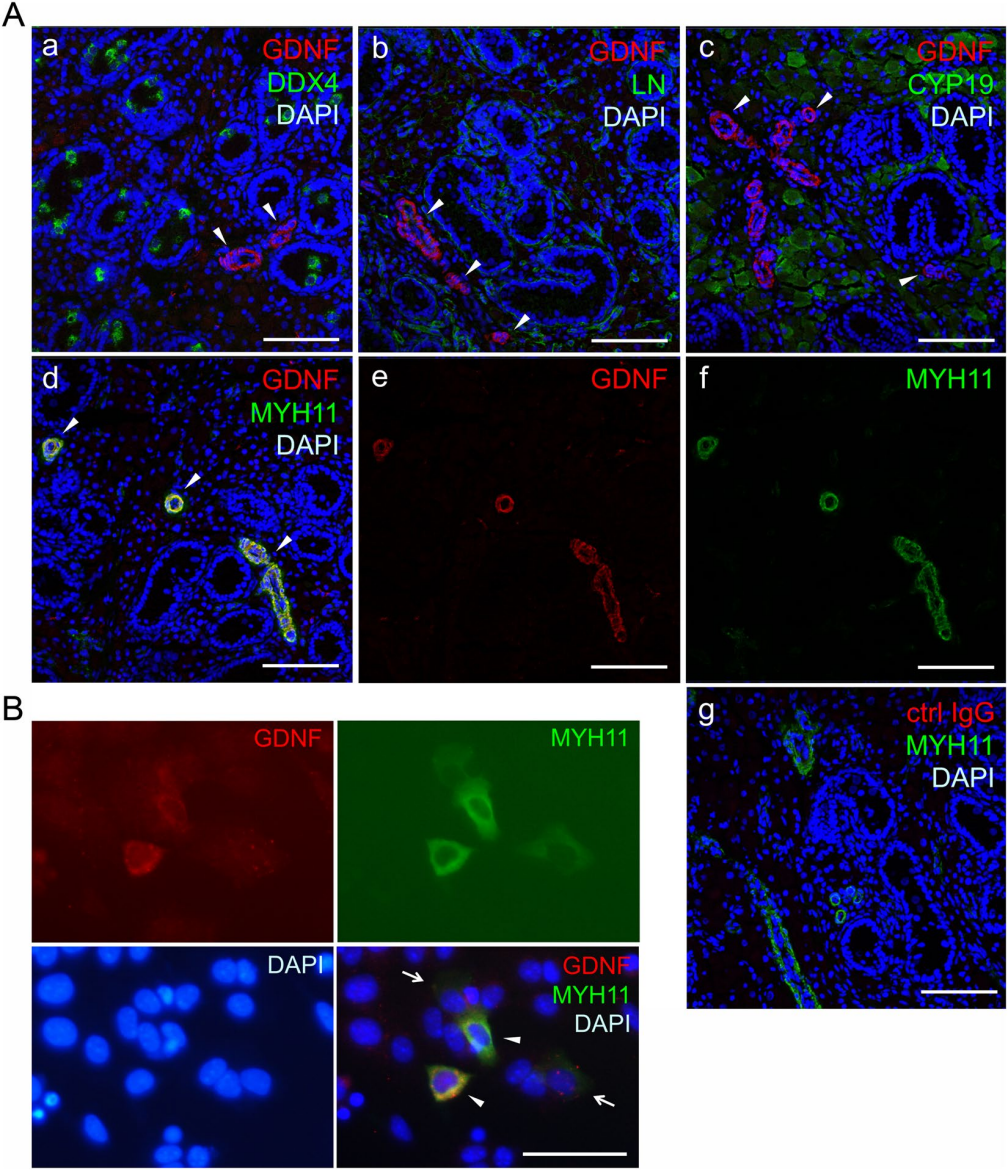
Expression analysis of pGDNF in postnatal porcine testes. (**A**) Immunohistochemistry of prepubertal porcine testis (36-day-old). Merged fluorescence images for GDNF and DDX4 (a), laminin (b), CYP19 (c), or MYH11(d) are shown. GDNF^+^ cells were detected only in the interstitial space (arrow head). DDX4^+^ cells (gonocytes/undifferentiated spermatogonia) were sparsely found within the lumen of the seminiferous tubules. The basement membrane of the seminiferous tubules was visualised by laminin staining. While Leydig cells identified by CYP19 staining were not GDNF^+^ (c), vascular smooth muscle cells identified by MYH11 staining were GDNF^+^ (d). Split fluorescence images of (d) are shown in (e,f). Rabbit control IgG (ctrl IgG) and MYH11 staining (g). Nuclei were counterstained with DAPI. Bars = 100 µm. (**B**) Immunocytochemistry of cultured porcine testis cells. Testicular cells prepared from postnatal pig (9-day-old) were cultured overnight followed by staining with anti-GDNF and anti-MYH11 antibodies. GDNF^+^ MYH11^+^ cells (arrow head) and GDNF^−^ MYH11^+^ cells (arrow) are indicated. Nuclei were counterstained with DAPI. Bars = 50 µm.

We next investigated GDNF expression in the postnatal porcine testis. Immunohistochemical analysis for the expression of GDNF, DDX4, and laminin (LN) showed that GDNF-expressing cells were localised in the interstitial space, but not inside the seminiferous tubules where DDX4^+^ gonocytes/undifferentiated spermatogonia were localized (Fig. 6Aa,b). In the interstitial space, GDNF^+^ cells appeared to be surrounding blood vessels, indicating that they are likely vascular smooth muscle cells. In addition, GDNF^+^ cells identified in the interstitial space did not express CYP19 (p450 aromatase), a marker for Leydig cells in porcine testes^38^ (Fig. 6Ac). To clarify whether vascular smooth muscle cells express GDNF, expression analysis of GDNF and MYH11, a marker for smooth muscle cells^39^ was performed. The result of immunohistochemistry indicated that the GDNF^+^ cells surrounding blood vessels expressed MYH11 (Fig. 6d–f). Another anti-GDNF antibody, which was raised by immunization with mouse GDNF, showed the same staining pattern (Fig. S1). To confirm these immunohistochemical results, immunocytochemical analysis of GDNF and MYH11 in one-day cultures of testicular cells from postnatal pigs was conducted. MYH11^+^ cells were identified in the culture, and some of them expressed GDNF (Fig. 6B). Collectively, these results indicated that GDNF is expressed by vascular smooth muscle cells in postnatal porcine testes.

**Figure 7:**
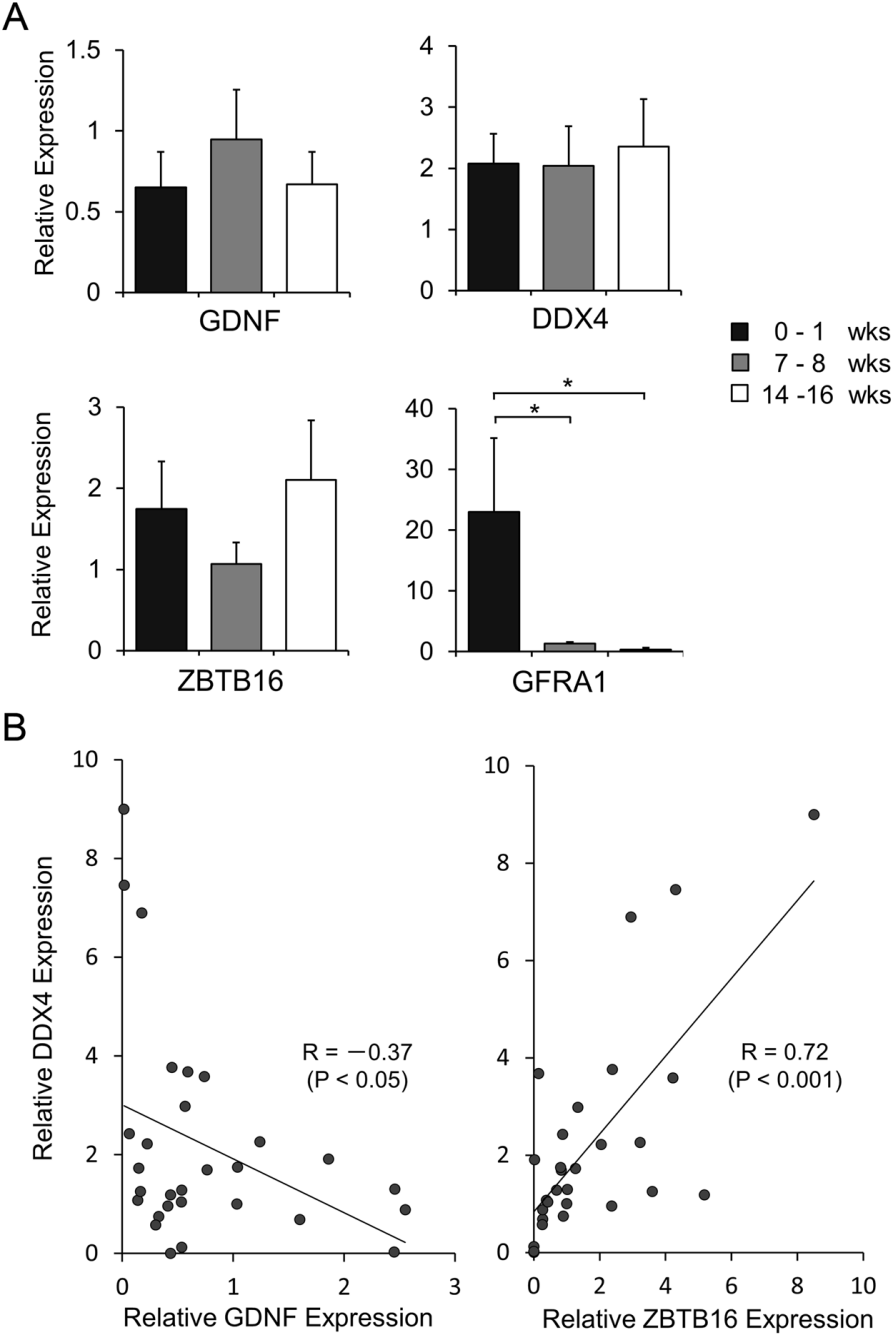
Transcriptional expression analysis of pGDNF in postnatal porcine testes. (**A**) Quantitative gene expression analysis of GDNF, DDX4, ZBTB16 and GFRA1 in prepubertal porcine testes. A total of 29 testes (7 from 0–1 weeks, 9 from 7–8 weeks and 13 from 14–16 weeks) were analysed for relative gene expression of the indicated genes by qRT-PCR. Values were normalised to the 18S ribosomal RNA expression levels. Data are presented as mean ± SEM. *P < 0.05. (**B**) Simple regression analysis between DDX4 expression and GDNF or ZBTB16 expression in individual testis samples. A negative correlation was found between DDX4 and GDNF (left), whereas a strong positive correlation was found between DDX4 and ZBTB16 (right).

GDNF mRNA expression in mouse and rat testes increases during the first week after birth, when gonocytes/undifferentiated spermatogonia give rise to SSCs and begin to proliferate^6,28^. Such spermatogonial proliferation occurs around 16 weeks in porcine testes^33^. If porcine SSCs or undifferentiated spermatogonia require GDNF for their proliferation, GDNF mRNA expression would increase during the same period as seen in rodents. Thus, we examined GDNF mRNA expression in 0–16-week-old porcine testes by quantitative RT-PCR (qRT-PCR) analysis. As shown in Fig. 7A, however, the mRNA expression level of pGDNF in the testes did not change between 0–16 weeks (P = 0.65). Although there was no increasing trend in the GDNF expression, DDX4 and the undifferentiated spermatogonia/SSCs marker, ZBTB16^34–36^, showed an increasing trend in the 14–16-week-old testes. In our previous immmunohistochemical analysis, ZBTB16 was expressed in majority of DDX4^+^ cells in postnatal porcine testes^34^. To confirm these correlations between GDNF, DDX4 and ZBTB16 expression patterns, analysis of individual testis samples was performed. The analysis indicated that expression of GDNF and DDX4 showed a weak, but significant negative correlation (R = −0.3702; P < 0.05) (Fig. 7B), whereas expression of ZBTB16 and DDX4 showed a strong positive correlation (R = 0.715833; P < 0.001) (Fig. 7B). These results indicate that GDNF expression is not associated with spermatogonial proliferation in the pig testes. Furthermore, because expression of GFRA1, the GDNF-binding receptor, was also increased during spermatogonial proliferation in mice^6^, GFRA1 expression was investigated in the pig testes. Although qRT-PCR analysis detected GFRA1 in 0–1-week-old testes, the expression was progressively decreased after the first week and became negligible in 14–16-week-old testes (Fig. 7A).

## Discussion

In the present study, we examined whether pGDNF has the ability to expand SSCs *in vitro* and evaluated its role in spermatogonial proliferation in porcine testes. We first determined the primary structure of pGDNF and generated its recombinant protein for biochemical and biological characterization. Our results demonstrated that the primary structure and biochemical characteristics of pGDNF were well-conserved with the human and rodent GDNFs that have been studied by several research groups^1,24–28^. In addition, our culture experiments demonstrated that the proliferative activity of pGDNF on murine SSCs was equivalent to that of hGDNF, which supports the expansion of murine SSCs both *in vivo* and *in vitro*^11,40^. However, the mitogenic effect of pGDNF on the porcine gonocytes/undifferentiated spermatogonia was negligible. Furthermore, pGDNF expression was localised to the interstitial vascular smooth muscle cells, and GFRA1 expression was progressively decreased in the testes after birth and became negligible when SSCs began to proliferate. These data strongly suggest that self-renewal and proliferation of porcine SSCs require extrinsic factors other than GDNF.

We initially isolated the full-length pGDNF cDNA from the testes, determined its sequence and generated recombinant pGDNF using mammalian cells. In the postnatal porcine testes, long and short forms of the pGDNF transcripts, which encode 211 and 185 amino acids respectively, were expressed. Compared with the previously reported GDNF sequences identified in human, rat, and mouse, the long and short transcripts correspond to pre-(α)pro GDNF and pre-(β)pro GDNF, respectively^24–28^. The two transcripts are generated by alternative splicing of the exon encoding the pro-domain and produce an identical mature GDNF (134 amino acids) by post-translational processing^30,41^. Although the sequences of pGDNF shared high homology (~90%) with those of human and rodent GDNF, the similarity was highest in case of bovine GDNF (95%). Pigs and cattle belong to the Laurasiatheria lineage, while humans and rodents belong to the Euarchontoglires lineage. The short transcript has been identified only in humans and rodents, but not in cattle. Therefore, to our knowledge, this is the first study reporting the identification of pre-(β)pro GDNF in species other than those belonging to Euarchontoglires.

Recent studies using neuronal cells have revealed that the intracellular localization and the secretory pathways of pre-(α)pro hGDNF and pre-(β)pro hGDNF are differently regulated^30,41^. It has been reported that (β)pro hGDNF is localised in the secretory granules of the regulated secretory pathway, while (α)pro hGDNF is localised mostly in the Golgi complex and is constitutively secreted^30^. Twenty five out of twenty six amino acids of the pro-domain, which is absent in pre-(β)pro GDNF, are identical between pig and human, and the pre-domain is completely conserved among human, rat, mouse, and pig. This suggests an evolutionary pressure to maintain the amino acid sequences of the pre- and pro-domains, which are likely to be involved in the regulation of the secretory pathways. Furthermore, our transfection experiments demonstrated that the intracellular and secreting forms of recombinant pGDNF, including the glycosylated forms were essentially identical to those of hGDNF. Intriguingly, the expression level of pGDNF by transfection of pre-(β)pro pGDNF cDNA into COS-1 cells was markedly reduced. Lower expression of GDNF from pre-(β)pro hGDNF cDNA than pre-(α)pro hGDNF cDNA has also been observed in CHO and neuronal cells^30,41^; however the reason behind this remains unclear. Collectively, all our data support that pGDNF retains high similarity to human and rodent GDNF, with regard to its primary structure and gene products.

As predicted, our *in vitro* culture experiments confirmed that the mitogenic activity of pGDNF and hGDNF on mouse SSCs was equivalent. Clump-forming germ cells enriched with SSCs proliferated in the presence of pGDNF at the same rate as that with hGDNF, and the transplantation assay for SSCs demonstrated that the germ cell clumps contained equal numbers of SSCs. In contrast, porcine gonocytes/undifferentiated spermatogonia did not respond to pGDNF. Although there are several reports attempting *in vitro* cultures of porcine gonocytes or type A spermatogonia in the presence of hGDNF^42,43^, no studies have demonstrated GDNF-dependent proliferation, as has been demonstrated in the studies of rodent and rabbit SSCs^10,14,19^. In addition, the culture medium used for pig germ cells contained foetal bovine serum (FBS) and a growth factor cocktail, including epidermal growth factor (EGF), leukaemia inhibitory factor (LIF) and fibroblast growth factor 2 (FGF2), which makes interpretation regarding growth factor-dependent proliferation of cells more difficult. Thus, no clear evidence demonstrating GDNF-dependent proliferation of porcine spermatogonia has been shown. In the present study, we used a serum-free medium (SFM)^11,31^ to directly evaluate the effect of GDNF on porcine gonocytes/undifferentiated spermatogonia. The SFM we developed has been proven valuable for assessing exogenous factors on mouse SSC proliferation^10,11^. It should be noted that some growth factors act on target cells in a species-specific manner. For example, human stem cell factor (SCF, also called KITL) shows no activity on rodent cells, but rodent SCF is active on human cells^44,45^. Thus, we generated recombinant pGDNF to rule out any possibility of hyporeactivity caused by species-specific activity of GDNF. Nonetheless, the mitogenic activity of pGDNF on porcine gonocytes/undifferentiated spermatogonia was negligible. This result strongly suggested that GDNF is not responsible for the proliferation of porcine gonocytes/undifferentiated spermatogonia. This was supported by the results of expression analysis of GDNF and GFRA1 by immunohistochemistry and qRT-PCR in porcine testes. In rodents, a peak expression of GDNF and GFRA1 mRNA was observed at the first week after birth when spermatogenesis initiated^6,28^. However, no increase was observed by the quantitative analysis of GDNF and GFRA1 mRNA expression in 14–16-week old pig testes, although some of porcine testis samples from 14–16-week old pigs partially started spermatogenesis. A previous study suggested that GFRA1 is expressed in a minor subpopulation of gonocytes/undifferentiated spermatogonia collected from 3-day-old pig testes^46^. In the study, only 30% of gonocytes/undifferentiated spermatogonia were found in the GFRA1^+^ cell fraction. Our qRT-PCR results showed that highest GFRA1 expression was seen during the first two weeks after birth, and then reduced in the following weeks. Thus, GFRA1 might be expressed only in the very early stage of germ cells in pigs. More importantly, no upregulation of GFRA1 expression was seen at the onset of spermatogenesis, during which the SSCs expand in number. Furthermore, although GDNF is expressed in Sertoli cells, myoid cells, and vascular smooth muscle cells in the mouse testis^6,39,47^, our immunohistochemical analysis of porcine testes demonstrated that GDNF expression was found only in vascular smooth muscle cells in the interstitial space, but not in Sertoli cells and myoid cells. Thus, these results indicate that remarkable species differences exist in the expression and distribution of GDNF in testes.

In conclusion, although pGDNF is structurally conserved and similar to hGDNF and rodent GDNF, all our data support that this factor does not play a central role in porcine SSC self-renewal and spermatogonial proliferation. Thus, the key mitogenic factor(s) that induce porcine SSC proliferation remains unclear. Several factors, including FGF2, SCF, CSF1, CXCL12 and WNTs have shown beneficial effects on spermatogonial proliferation in mice or humans^11,48–51^. A single or combination of these factors might be able to stimulate the proliferation of porcine spermatogonia. It is extremely important to identify factors essential for the *in vitro* expansion of porcine SSCs, because the development of long-term culture systems is crucial to understand their basic biology and exploit new technologies for germline modification, which contribute to the advancement of agricultural and biomedical sciences^52^. In addition, no unequivocal evidence showing that GDNF is the essential factor for SSC self-renewal has been demonstrated in any mammals other than rodents and rabbits^53^. In particular, *ex vivo* expansion of human SSCs is important to establish the foundation for human germ cell research and for development of new reproductive technologies. Although a subpopulation of human spermatogonia expresses GFRA1^54^, it is not clear whether the GFRA1^+^ spermatogonia can proliferate in a GDNF-dependent manner^55^. Thus, elucidation of exogenous factors supporting proliferation of porcine SSCs are of great interest for studies on human SSCs. Further investigation of factors essential for the self-renewal of porcine SSCs will provide new insights into mammalian germline stem cell biology.

## Materials and Methods

All experimental procedures were approved by and conducted in accordance with the Guidelines for Institutional Laboratory Animal Care and Use of the School of Veterinary Medicine at Kitasato University. Supplemental methods are available in Supplemental Experimental Procedures. Primer sequences used for PCR are listed in Table S1. Antibodies used for immunostaining and immunoblot analysis are listed in Table S2. Unless otherwise stated, all chemicals and reagents were from Sigma-Aldrich (St. Louis, MO, USA).

### cDNA cloning of porcine GDNF

Total RNA was extracted with TRIzol (Thermo Fisher Scientific, Waltham, MA, USA) from 7-day-old pig (Duroc × Large white) testes followed by RNeasy column purification (Qiagen, Hilden, Germany)^34^. cDNA was synthesised from 5 µg total RNA by oligo-dT priming using the SuperScript III First-Strand Synthesis System (Thermo Fisher Scientific). To clone the complete pGDNF cDNA by RT-PCR, a primer set (pGDNF 5UT-Fw, pGDNF 3UT-Rv) was designed with reference to the 5′- and 3′-untranslated regions of human, mouse, cattle, and dog GDNF mRNA (GenBank accession number L19063.1, U37459.1, XM_615361.4, and XM_546342.2, respectively). PCR amplification consisted of 1 cycle of 5 min at 94 °C followed by 30 cycles each of 30 s at 94 °C, 2 s at 55 °C, and 30 s at 74 °C, and was performed using Taq DNA polymerase (Takara Bio, Shiga, Japan). The amplified product was cloned into pCR-Blunt II-TOPO (Thermo Fisher Scientific) and sequenced. Based on the sequences obtained, a second primer set (pGDNF comp-Fw, pGDNF comp-Rv) for RT-PCR was designed to amplify the complete coding region of pGDNF cDNA. The forward and reverse primers were placed in the 5′- and 3′-untranslated regions of the GDNF genes, respectively. PCR amplification using the second primer set consisted of 1 cycle of 5 min at 94 °C, followed by 30 cycles, each of 10 s at 98 °C, 5 s at 55 °C, and 60 s at 72 °C, and was performed using Prime STAR polymerase (Takara Bio). The PCR products were cloned into pCR-Blunt II-TOPO for sequencing and subsequently subcloned into the mammalian-expression plasmid, pEPI-CAG^34^, resulting in pEPI-CAG-pGDNF. The hGDNF coding cDNA (a kind gift from Dr. H. Sariola, University of Helsinki, Finland) was also subcloned into the pEPI-CAG, resulting in pEPI-CAG-hGDNF.

### Transfection

COS-1 cells cultured in 60 mm dishes were transfected with 2 µg of pEPI-CAG-pGDNF or pEPI-CAG-hGDNF by the DEAE-dextran/chloroquine method as described previously^34,56^. Twenty-four hours after transfection, the cells were replated on a 12-well plate. On the next day, the medium was replaced with serum-free RPMI-1640. After two days of incubation, the supernatants and the transfected cells were separately collected for western blot analysis. To establish cell lines constitutively expressing recombinant pGDNF or hGDNF, pEPI-CAG-pGDNF or pEPI-CAG-hGDNF was transfected into HEK293 cells, respectively, using Lipofectamine 2000 (Thermo Fisher Scientific). Following G418-selection, high-expressing clones were selected. After the cloned cell lines were expanded in a serum-supplemented growth medium (DMEM with 10% FBS), they were replated on 12-wells plates coated with 0.1% gelatine at a density of 10^5^ cells/well, and then cultured in SFM^10,11,31^ that consisted of minimum essential medium α (Thermo Fisher Scientific) supplemented with 0.2% bovine serum albumin (BSA, A7030; Sigma-Aldrich), 5 µg/ml insulin (Wako Pure Chemicals, Osaka, Japan), 10 µg/ml iron-saturated transferrin, 30 nM Na_2_SeO_3_, 60 µM Putrescine, 7.6 µeq/L free fatty acid mixture, 50 µM 2-mercaptoethanol, 2 mM GlutaMAX (Thermo Fisher Scientific), 10 mM HEPES, and 50 units/ml Penicillin-50 µg/ml Streptomycin (Thermo Fisher Scientific). After 3 days of incubation, the culture medium was collected, centrifuged at 600× *g* for 5 min at 4 °C, filtered through a 0.2 μm filter, and then used for SSC cultures.

### Cell Preparation

Porcine testes were obtained after castration of 3- to 117-day-old (0- to 16-week-old) pigs housed in Kitasato University Field Science Center, Towada Experimental Farm. Porcine testicular cell suspensions were prepared as described previously^34^. Briefly, after removing the *tunica albuginea*, testis tissues were minced and digested with 1 mg/mL type IV collagenase (C5138; Sigma-Aldrich) at 37 °C for 15 min followed by incubation with 0.25% trypsin-EDTA (Thermo Fisher Scientific) and 7 mg/mL DNase at 37 °C for 15 min. Subsequently the testicular cell suspension was treated with red blood cell lysis buffer (0.15 M NH_4_Cl and 0.01 M KHCO_3_), overlaid with 20% Percoll solution, and centrifuged at 600 × *g* for 5 min at 4 °C to remove the red blood cells and debris. The cell pellet was resuspended in SFM and subjected to enrichment of gonocytes/undifferentiated spermatogonia by differential plating as described previously with some modifications^34^. The testicular cells were placed on gelatine-coated culture dishes with SFM and incubated in a 5% CO_2_ incubator at 37 °C for 24 h. Non-adherent and weakly attached cells were collected and cultured on gelatine-coated culture dishes for another 24 h. The floating and weakly attached cells were collected and used as gonocytes/undifferentiated spermatogonia-enriched cell populations. Enrichment was confirmed by western blot analysis and counting the cell number of gonocytes/undifferentiated spermatogonia in the fractionated cell suspensions.

### Cell proliferation assay and immunocytochemistry

For cell proliferation assay, the gonocytes/undifferentiated spermatogonia-enriched cells were seeded at a density of 1–5 × 10^5^ cells/well in 24-well plates coated with Matrigel (Corning, Corning, NY, USA). The Matrigel was diluted 60-fold with SFM. The cells were cultured in SFM in the presence or absence of pGDNF (30 μL/mL) for 6 days. The medium was changed every other day. To identify proliferating cells in culture, cultured cells were immunocytochemically stained with anti-Ki67 antibody or labelled with EdU incorporation. For immunocytochemistry, the cells cultured for 6 days were fixed with 2% paraformaldehyde (PFA) and stained with mouse anti-Ki67 antibody (Leica Biosystems, Newcastle Upon Tyne, UK) and rabbit anti-human DDX4 antibody (Abcam, Cambridge, MA, USA), followed by staining with Alexa Flour 488-conjugated goat anti-mouse IgG (Thermo Fisher Scientific) and Alexa Flour 568-conjugated goat anti-rabbit IgG (Thermo Fisher Scientific) for 60 min at room temperature (~25 °C). After antibody staining, nuclei were counter-stained with 0.5 µg/mL 4′,6-diamidino-2-phenylindole (DAPI; Thermo Fisher Scientific). For EdU labelling, EdU was added at day 5 in the 24-well plate culture to a concentration of 10 µM. After 24 h, the cultures were fixed with 2% PFA, and incorporated EdU was detected by the Click-iT EdU Imaging Kit (Thermo Fisher Scientific). Subsequently, the cells were stained with rabbit anti-human DDX4 antibody followed by staining with Alexa Flour 568-conjugated goat anti-rabbit IgG and DAPI for nuclear counterstaining. For EdU labeling of unenriched cells, freshly isolated cells from testes were cultured for 8 days at a density of 0.5 × 10^5^ cells/well in 24-well plates coated with Matrigel. EdU was added at day 7 in culture to a concentration of 10 µM. After 24 h, detection of incorporated EdU and DDX4 antibody staining in the cultures were performed as described above. To measure proliferation of gonocytes/undifferentiated spermatogonia in culture, Ki67^+/−^ DDX4^+^ or EdU^+/−^ DDX4^+^ cells were counted using a fluorescence microscope (DMRE; Leica Microsystems). For immunocytochemical analysis of GDNF and MYH11 in porcine testicular cells, unenriched testicular cells from postnatal testes were seeded at a density of 2 × 10^5^ cells/well in 24-well plates coated with Matrigel as described above. Next day, cultured cells were fixed with 2% PFA and stained with rabbit anti-mouse GDNF antibody (Abcam) and mouse anti-human MYH11 antibody (Santa Cruz Biotechnology, Santa Cruz, CA, USA), followed by staining with Alexa Flour 568-conjugated goat anti-rabbit IgG (Thermo Fisher Scientific) and Alexa Flour 488-conjugated goat anti-mouse IgG (Thermo Fisher Scientific) for 60 min at room temperature. After antibody staining, nuclei were counter-stained with DAPI, and stained cells were analysed using a fluorescence microscope (DMRE; Leica Microsystems).

### SSC culture

Mouse SSC cultures were established as described previously^10,31,57^ with minor modifications. In brief, murine testicular cells were prepared from 5-day-old GFP mice (C57BL/6-Tg (CAG-EGFP), Japan SLC, Shizuoka, Japan) by enzymatic digestion using trypsin-EDTA, and cultured on SNL 76/7 STO cell (a kind gift from Dr. A. Bradley, Baylor College of Medicine, USA) feeders in SFM with 10 ng/mL hGDNF (R&D Systems, Minneapolis, MN, USA) and 0.5 ng/mL human FGF2 (Corning). In some experiments, 30 μL/mL of pGDNF were used instead of hGDNF. One day after seeding, weakly attached spermatogonia were collected and cultured on fresh feeders. Medium was changed every other day, and the cells were subcultured every 5–7 days as described previously^31^.

### Transplantation

Cell suspensions of germ cell clumps from GFP mice were transplanted into recipient C57BL/6 mouse testes via efferent duct injection^58^. Recipient mice were treated with 44 mg/kg Busulfan at least six weeks prior to transplantation, and 8 μL of 1.5 × 10^6^ cells/mL were then transplanted into each testis. Two months after transplantation, the testes were analysed using a fluorescent stereomicroscope (SZX16; Olympus, Tokyo, Japan). Spermatogenic colonies consisting of GFP^+^ cells were counted and normalised to 1 × 10^5^ cells transplanted into the recipient testes. For the histology, recipient testes were embedded in paraffin wax, sectioned and stained with hematoxylin and eosin.

### Immunohistochemistry

Porcine testis tissue fragments were embedded in an OTC compound (Sakura Finetechnical, Tokyo, Japan), frozen in liquid nitrogen, and stored at −80 °C until sectioning^34^. Frozen sections were cut into 6-μm-thick slices, placed onto glass slides, and fixed in acetone for 30 s. After 20 min of incubation with blocking solution (4% goat serum, 1% BSA, 0.1% cold fish skin gelatine, 0.1% Triton X-100, 0.05% Tween 20, and 0.05% sodium azide in phosphate buffered saline), the sections were stained with anti-human GDNF antibody (Santa Cruz Biotechnology) or anti-mouse GDNF antibody (Abcam) and anti-DDX4 antibody (Abcam) anti-Laminin antibody (Leica Biosystems), anti-CYP19 antibody (Santa Cruz Biotechnology), or anti-MYH11 antibody at 4 °C overnight. Normal rabbit IgG (Wako Pure Chemicals) and isotype control mouse IgG_1_ (Biolegend, San Diego, CA, USA) were used as negative controls. The sections were then stained with Alexa Flour 488-conjugated anti-mouse IgG (Thermo Fisher Scientific) and Alexa Flour 568-conjugated anti-rabbit IgG (Thermo Fisher Scientific) for 60 min at room temperature, followed by nuclear counterstaining with DAPI and analysis using a laser scanning confocal microscope (LSM710; Carl Zeiss, Oberkochen, Germany).

### Statistical analysis

All data are presented as mean ± standard error of mean (SEM) and their simple comparisons were performed using Student’s t-test. Multiple comparisons between the groups were analysed by the Tukey-Kramer test. The strength of a relationship was measured by Pearson’s correlation coefficient test.

### Additional code

Sequencing data is available from the DNA Data Bank of Japan (DDBJ) under the accession number AB675652-AB675653.

## Electronic supplementary material

Supplementary Information

## References

[1] Lin LF, Doherty DH, Lile JD, Bektesh S, Collins F (1993). GDNF: a glial cell line-derived neurotrophic factor for midbrain dopaminergic neurons. Science.

[2] Golden JP, DeMaro JA, Osborne PA, Milbrandt J, Johnson EM (1999). Expression of Neurturin, GDNF, and GDNF Family-Receptor mRNA in the Developing and Mature Mouse. Exp. Neurol..

[3] Moore MW (1996). Renal and neuronal abnormalities in mice lacking GDNF. Nature.

[4] Pichel JG (1996). Defects in enteric innervation and kidney development in mice lacking GDNF. Nature.

[5] Sanchez MP (1996). Renal agenesis and the absence of enteric neurons in mice lacking GDNF. Nature.

[6] Meng X (2000). Regulation of cell fate decision of undifferentiated spermatogonia by GDNF. Science.

[7] Tegelenbosch RA, de Rooij DG (1993). A quantitative study of spermatogonial multiplication and stem cell renewal in the C3H/101 F1 hybrid mouse. Mutat. Res..

[8] Brinster RL, Avarbock MR (1994). Germline transmission of donor haplotype following spermatogonial transplantation. Proc. Natl. Acad. Sci. USA.

[9] Brinster RL, Zimmermann JW (1994). Spermatogenesis following male germ-cell transplantation. Proc. Natl. Acad. Sci. USA.

[10] Kubota H, Avarbock MR, Brinster RL (2004). Culture Conditions and Single Growth Factors Affect Fate Determination of Mouse Spermatogonial Stem Cells. Biol. Reprod..

[11] Kubota H, Avarbock MR, Brinster RL (2004). Growth factors essential for self-renewal and expansion of mouse spermatogonial stem cells. Proc. Natl. Acad. Sci. USA.

[12] Ogawa T, Dobrinski I, Avarbock MR, Brinster RL (1999). Xenogeneic spermatogenesis following transplantation of hamster germ cells to mouse testes. Biol. Reprod..

[13] Clouthier DE, Avarbock MR, Maika SD, Hammer RE, Brinster RL (1996). Rat spermatogenesis in mouse testis. Nature.

[14] Ryu BY, Kubota H, Avarbock MR, Brinster RL (2005). Conservation of spermatogonial stem cell self-renewal signaling between mouse and rat. Proc. Natl. Acad. Sci. USA.

[15] Dobrinski I, Avarbock MR, Brinster RL (2000). Germ cell transplantation from large domestic animals into mouse testes. Mol. Reprod. Dev..

[16] Dobrinski I, Avarbock MR, Brinster RL (1999). Transplantation of germ cells from rabbits and dogs into mouse testes. Biol. Reprod..

[17] Nagano M, McCarrey JR, Brinster RL (2001). Primate spermatogonial stem cells colonize mouse testes. Biol. Reprod..

[18] Nagano M, Patrizio P, Brinster RL (2002). Long-term survival of human spermatogonial stem cells in mouse testes. Fertil. Steril..

[19] Kubota H, Wu X, Goodyear SM, Avarbock MR, Brinster RL (2011). Glial cell line-derived neurotrophic factor and endothelial cells promote self-renewal of rabbit germ cells with spermatogonial stem cell properties. The FASEB Journal.

[20] Nagano M (2001). Transgenic mice produced by retroviral transduction of male germ-line stem cells. Proc. Natl. Acad. Sci. USA.

[21] Brinster RL (2002). Germline stem cell transplantation and transgenesis. Science.

[22] Culty M (2013). Gonocytes, from the Fifties to the Present: Is There a Reason to Change the Name?. Biol. Reprod..

[23] McCarrey JR (2013). Toward a More Precise and Informative Nomenclature Describing Fetal and Neonatal Male Germ Cells in Rodents. Biol. Reprod..

[24] Schaar DG (1994). Multiple Astrocyte Transcripts Encode Nigral Trophic Factors in Rat and Human. Exp. Neurol..

[25] Grimm L (1998). Analysis of the human GDNF gene reveals an inducible promoter, three exons, a triplet repeat within the 3′-UTR and alternative splice products. Hum. Mol. Genet..

[26] Matsushita N, Fujita Y, Tanaka M, Nagatsu T, Kiuchi K (1997). Cloning and structural organization of the gene encoding the mouse glial cell line-derived neurotrophic factor, GDNF1. Gene.

[27] Suter-Crazzolara C, Unsicker K (1994). GDNF is expressed in two forms in many tissues outside the CNS. Neuroreport.

[28] Trupp M (1995). Peripheral expression and biological activities of GDNF, a new neurotrophic factor for avian and mammalian peripheral neurons. The Journal of Cell Biology.

[29] Oh-hashi K, Ito M, Tanaka T, Hirata Y, Kiuchi K (2009). Biosynthesis, processing, and secretion of glial cell line-derived neurotrophic factor in astroglial cells. Mol. Cell. Biochem..

[30] Lonka-Nevalaita L (2010). Characterization of the Intracellular Localization, Processing, and Secretion of Two Glial Cell Line-Derived Neurotrophic Factor Splice Isoforms. The Journal of Neuroscience.

[31] Kubota H, Brinster RL (2008). Culture of rodent spermatogonial stem cells, male germline stem cells of the postnatal animal. Methods Cell Biol..

[32] Kubota H, Avarbock MR, Brinster RL (2003). Spermatogonial stem cells share some, but not all, phenotypic and functional characteristics with other stem cells. Proc. Natl. Acad. Sci. USA.

[33] Malmgren L, Rodriguez-Martinez H, Einarsson S (1996). Attainment of spermatogenesis in Swedish cross-bred boars. Zentralbl. Veterinarmed. A.

[34] Kakiuchi K (2014). Cell-Surface DEAD-Box Polypeptide 4-Immunoreactive Cells and Gonocytes Are Two Distinct Populations in Postnatal Porcine Testes. Biol. Reprod..

[35] Luo J, Megee S, Dobrinski I (2009). Asymmetric Distribution of UCH-L1 in Spermatogonia Is Associated With Maintenance and Differentiation of Spermatogonial Stem Cells. J. Cell. Physiol..

[36] Manku G, Culty M (2015). Mammalian gonocyte and spermatogonia differentiation: recent advances and remaining challenges. Reproduction.

[37] Kim B-G (2010). Enrichment of Testicular Gonocytes and Genetic Modification Using Lentiviral Transduction in Pigs. Biol. Reprod..

[38] Mutembei HM, Pesch S, Schuler G, Hoffmann B (2005). Expression of Oestrogen Receptors α and β and of Aromatase in the Testis of Immature and Mature Boars. Reproduction in Domestic Animals.

[39] Chen S-R, Liu Y-X (2016). Myh11-Cre is not limited to peritubular myoid cells and interaction between Sertoli and peritubular myoid cells needs investigation. Proceedings of the National Academy of Sciences.

[40] Yomogida K, Yagura Y, Tadokoro Y, Nishimune Y (2003). Dramatic expansion of germinal stem cells by ectopically expressed human glial cell line-derived neurotrophic factor in mouse Sertoli cells. Biol. Reprod..

[41] Wang Y (2008). GDNF isoform affects intracellular trafficking and secretion of GDNF in neuronal cells. Brain Res..

[42] Zheng Y (2014). Spermatogonial stem cells from domestic animals: progress and prospects. Reproduction.

[43] González R, Dobrinski I (2015). Beyond the Mouse Monopoly: Studying the Male Germ Line in Domestic Animal Models. ILAR Journal.

[44] Martin FH (1990). Primary structure and functional expression of rat and human stem cell factor DNAs. Cell.

[45] Lev S, Yarden Y, Givol D (1992). Dimerization and activation of the kit receptor by monovalent and bivalent binding of the stem cell factor. J. Biol. Chem..

[46] Lee KH (2013). Characterization of GFRα-1-Positive and GFRα-1-Negative Spermatogonia in Neonatal Pig Testis. Reproduction in Domestic Animals.

[47] Chen L-Y, Willis WD, Eddy EM (2016). Targeting the Gdnf Gene in peritubular myoid cells disrupts undifferentiated spermatogonial cell development. Proceedings of the National Academy of Sciences.

[48] Takase HM, Nusse R (2016). Paracrine Wnt/β-catenin signaling mediates proliferation of undifferentiated spermatogonia in the adult mouse testis. Proceedings of the National Academy of Sciences.

[49] Oatley JM, Oatley MJ, Avarbock MR, Tobias JW, Brinster RL (2009). Colony stimulating factor 1 is an extrinsic stimulator of mouse spermatogonial stem cell self-renewal. Development.

[50] Yang QE, Kim D, Kaucher A, Oatley MJ, Oatley JM (2013). CXCL12-CXCR4 signaling is required for the maintenance of mouse spermatogonial stem cells. J. Cell Sci..

[51] Tu J (2007). Stem cell factor affects fate determination of human gonocytes *in vitro*. Reproduction.

[52] Kubota H, Brinster RL (2006). Technology insight: *In vitro* culture of spermatogonial stem cells and their potential therapeutic uses. Nature Clinical Practice Endocrinology & Metabolism.

[53] Kubota, H. & Brinster, R. L. Transplantation and culture of spermatogonial stem cells. In *The Biology of Mammalian Spermatogonia* (eds Jon Oatley & Michael Griswold) 271–300 (Springer New York, 2017).

[54] Di Persio S (2017). Spermatogonial kinetics in humans. Development.

[55] Medrano JV, Rombaut C, Simon C, Pellicer A, Goossens E (2016). Human spermatogonial stem cells display limited proliferation *in vitro* under mouse spermatogonial stem cell culture conditions. Fertil. Steril..

[56] Kubota H (1990). Identification and gene cloning of a new phosphatidylinositol-linked antigen expressed on mature lymphocytes. Down-regulation by lymphocyte activation. J. Immunol..

[57] Kubota H, Avarbock MR, Schmidt JA, Brinster RL (2009). Spermatogonial stem cells derived from infertile Wv/Wv mice self-renew *in vitro* and generate progeny following transplantation. Biol.Reprod..

[58] Ogawa T, Arechaga JM, Avarbock MR, Brinster RL (1997). Transplantation of testis germinal cells into mouse seminiferous tubules. Int. J. Dev. Biol..

