# Supplementary material for: Conserved and non-conserved characteristics of porcine glial cell line-derived neurotrophic factor expressed in the testis
Source: Sci Rep. 2018 May 16;8:7656. doi: 10.1038/s41598-018-25924-5 (PMC5955883; doi:10.1038/s41598-018-25924-5)
Supplement: Supplementary file 1 — Supplementary Information [file 41598_2018_25924_MOESM1_ESM.pdf]

SUPPLEMENTARY INFORMATION

Conserved and non-conserved characteristics of porcine glial cell line-derived neurotrophic factor expressed in the testis

Kazue Kakiuchi[1], Kazumi Taniguchi[2], Hiroshi Kubota[1*]

[1] Laboratory of Cell and Molecular Biology, School of Veterinary Medicine, Kitasato University, Towada, Aomori, Japan.

[2] Laboratory of Veterinary Anatomy, School of Veterinary Medicine, Kitasato University, Towada, Aomori, Japan.

[*]Correspondence and request for materials should be addressed to H.K. (email: hiroshi@vmas.kitasato-u.ac.jp)

- Supplemental Experimental Procedures
- Table S1
- Table S2
- Figure S1

Supplemental Experimental Procedures

Western blot analysis. COS-1 cells transfected with plasmids and testicular cells were lysed with RIPA buffer (2% SDS, 20 mM Tris-HCl pH 6.7, 5 mM EDTA, and 80 mM DTT), and the protein concentration was determined by the Lowly method[1]. Cell lysates (10 μg) and supernatants (10 μL) were separated by 10% SDS–PAGE and transferred to polyvinylidene difluoride (PVDF) membranes. The membranes were blocked in 2% skim milk in phosphate buffered saline for 1 h at room temperature, and then stained with rabbit anti-human GDNF antibody (Santa Cruz Biotechnology). Mouse anti-human α-tubulin (Santa Cruz Biotechnology) was used for validation of protein amount. Horseradish peroxidase-conjugated donkey anti-rabbit IgG (GE Healthcare, Piscataway, NJ, USA) or sheep anti-mouse IgG (GE Healthcare) was used as the secondary antibody, and signals were detected using the ECL advance system (GE Healthcare). For *N*-glycosylation analysis, cell lysates and supernatants were incubated with PNGase F (New England Biolabs, Ipswich, MA, USA) for 1 h at 37°C before SDS-PAGE. The amount of GDNF in the supernatants was evaluated by comparing the band density of purified recombinant hGDNF (R&D systems) using the ImageJ software (NIH, Bethesda, MD, USA). To confirm enrichment of gonocytes/undifferentiated spermatogonia, testis cell lysates (5 μg) were separated by 5–20% e-PAGEL (ATTO, Tokyo, Japan) and transferred to PVDF membranes. After blocking with 2% skim milk, the membranes were stained with rabbit anti-human DDX4 antibody (Abcam), rabbit anti-human UCHL1 antibody (Enzo, NY, USA), or mouse anti-GAPDH (Wako Pure Chemicals) for validation of protein amount, followed by staining with secondary antibodies as described above.

Flow cytometry. Single cell suspensions of murine germ cell clumps were stained with R-phycoerythrin (PE)-conjugated anti-THY1 (Biolegend), PE-conjugated anti-ITGA6 (Biolegend), PE-conjugated anti-ITGAV (Biolegend), or PE-conjugated anti-KIT (Biolegend)[2]. The stained cells were analysed by FC500 flow cytometer (Beckman Coulter, Brea, CA, USA).

Quantitative RT-PCR. Total RNA was extracted from porcine testes, and cDNA was synthesised as described in Materials and Methods. qRT-PCR was performed using the StepOnePlus and the SYBR Green PCR master mix (Thermo Fisher Scientific)[1]. Relative gene expression was determined by normalizing the level of target gene expression against the 18s rRNA expression by using the 2-ΔΔCT method.

References

1 Kakiuchi, K. *et al.* Cell-surface DEAD-box polypeptide 4-immunoreactive cells and gonocytes are two distinct populations in postnatal porcine testes. *Biol. Reprod.* **90**, 82, 81-11, doi:10.1095/biolreprod.113.114405 (2014).

2 Kubota, H., Avarbock, M. R. & Brinster, R. L. Growth factors essential for self-renewal and expansion of mouse spermatogonial stem cells. *Proc. Natl. Acad. Sci. U. S. A.* **101**, 16489-16494 (2004).

Table S1. Primer sequences used in cDNA cloning and qRT-PCR

**Primer**	**Sequence (5' to 3')**
cDNA cloning	
pGDNF 5UT-Fw	GSCDCYTGGAGTTAATGTCC
pGDNF 3UT-Rv	TTRCTGGGAACCTTGGTCCC
pGDNF comp-Fw	GCCGCTGGACGGGACTCTAAG
pGDNF comp-Rv	GGGAACCTTTGGTCCCGTTC
qRT-PCR	
pGDNF Fw	TGTCGTGGCTGTCTGCCTGGTG
pGDNF Rv	GGCGCCTCGGGAGGCCTCTTA
pDDX4 Fw	GCGTACGGGTCGCTGTGGAAA
pDDX4 Rv	CACGCAGGAACATCCTGTTGAGCAT
pZBTB16 Fw	AAAGCGGTTCCTGGATAGTTTG
pZBTB16 Rv	GGTCTGCCTGTGTGTCTCC
pGFRA1 Fw	ATAGACTCTAGTAGCCTCAG
pGFRA1 Rv	AGGGACTTGTTCTTGACC
p18S Fw	CCCGACGTGACTGCTCGGTG
p18S Rw	CTCGACCGAGGGCACAAGCG

Table S2. Antibodies used for immunocytochemistry, immunohistochemistry, flow cytometry and western blot analysis

**Antibody Name (Label)**	**Source**	**Dilution**
Primary antibody		
mouse anti-Ki67	NCL-L-Ki67-MM1; Leica Biosystems	1:100
rabbit anti-human DDX4	ab13840; Abcam	1:2000–10000
rabbit anti-human GDNF	sc-328; Santa Cruz Biotechnology	1:10000
mouse anti-human α-tubulin	sc-5286; Santa Cruz Biotechnology	1:10000
rabbit anti-human UCHL1	ADI-905-520-1; Enzo	1:5000
mouse anti-human GAPDH	010-25521; Wako Pure Chemicals	1:10000
mouse anti-human Laminin	NCL-LAMININ; Leica Biosystems	1:200
rabbit anti-human GDNF	sc-328; Santa Cruz Biotechnology	1:160
rabbit anti-mouse GDNF	ab-18956; Abcam	1:400
mouse anti-human MYH11	sc-65734; Santa Cruz Biotechnology	1:100
mouse anti-human CYP19	sc-374176; Santa Cruz Biotechnology	1:100
rat anti-mouse THY1 (PE)	105307; Biolegend	1:400
rat anti-mouse ITGA6 (PE)	313611; Biolegend	1:400
rat anti-mouse ITGAV (PE)	104105; Biolegend	1:400
rat anti-mouse KIT (PE)	105807; Biolegend	1:400
isotype control mouse IgG_1	401401; Biolegend	1:250
normal rabbit IgG	148-09551; Wako Pure Chemical	1.25 μg/mL
Secondary antibody		
goat anti-mouse IgG (Alexa 488)	A21131; Thermo Fisher Scientific	1:1000
goat anti-rabbit IgG (Alexa 568)	A11011; Thermo Fisher Scientific	1:1000
donkey anti-rabbit IgG (Horseradish peroxidase)	NA934; GE Healthcare	1:10000–20000
sheep anti-mouse IgG (Horseradish peroxidase)	NA931; GE Healthcare	1:10000–20000

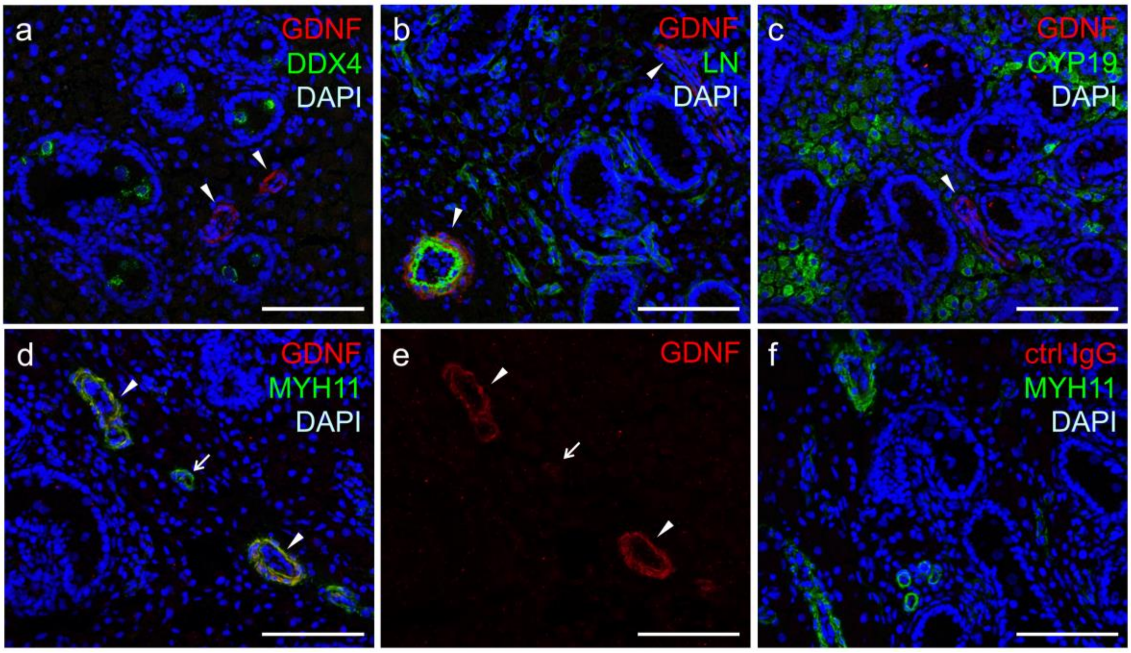

Figure S1. Expression analysis of pGDNF in prepubertal porcine testis.

Immunohistochemistry of frozen sections from a 36-day-old porcine testis stained with anti-mouse GDNF antibody and anti-DDX4 (**a**), anti-laminin (**b**), anti-CYP19 (**c**), or anti-MYH11 antibody (**d**). (**e**) Split fluorescent image for GDNF of (**d**). GDNF^{+} cells (arrow head) and GDNF^{-} MYH11^{+} cells (arrow) are indicated. (**f**) Staining with rabbit control IgG (ctrl IgG) and anti-MYH11 antibody. Ctrl IgG did not react with MHY11^{+} cells. Nuclei were counterstained with DAPI. Bars = 100 µm.
